# OCT Findings in MINOCA

**DOI:** 10.3390/jcm10132759

**Published:** 2021-06-23

**Authors:** Krzysztof Bryniarski, Pawel Gasior, Jacek Legutko, Dawid Makowicz, Anna Kedziora, Piotr Szolc, Leszek Bryniarski, Pawel Kleczynski, Ik-Kyung Jang

**Affiliations:** 1 Jagiellonian University Medical College, Institute of Cardiology, Department of Interventional Cardiology, John Paul II Hospital, 31-202 Krakow, Poland; kbrynia@gmail.com (K.B.); jacek.legutko@uj.edu.pl (J.L.); piotr.szolc4@gmail.com (P.S.); kleczu@interia.pl (P.K.); 2 Division of Cardiology and Structural Heart Diseases, Medical University of Silesia, 40-635 Katowice, Poland; p.m.gasior@gmail.com; 3 Interventional Cardiology, Electrotherapy and Angiology Department, John Paul II Hospital, 38-400 Krosno, Poland; david1990@onet.pl; 4 Department of Cardiovascular Surgery and Transplantation, John Paul II Hospital, 31-202 Krakow, Poland; kdzra.a@gmail.com; 5 2nd Department of Cardiology and Cardiovascular Interventions, University Hospital, Institute of Cardiology, Jagiellonian University Medical College, 31-501 Krakow, Poland; l_bryniarski@poczta.fm; 6 Cardiology Division, Massachusetts General Hospital, Harvard Medical School, 55 Fruit Street|GRB 800, Boston, MA 02114, USA; 7 Department of Cardiology, School of Medicine, Kyung Hee University, Dongdaemoon-gu, Seoul 130-701, Korea

**Keywords:** cardiovascular disease, acute myocardial infarction, intravascular imaging

## Abstract

Myocardial infarction with non-obstructive coronary artery disease (MINOCA) is a working diagnosis for patients presenting with acute myocardial infarction without obstructive coronary artery disease on coronary angiography. It is a heterogenous entity with a number of possible etiologies that can be determined through the use of appropriate diagnostic algorithms. Common causes of a MINOCA may include plaque disruption, spontaneous coronary artery dissection, coronary artery spasm, and coronary thromboembolism. Optical coherence tomography (OCT) is an intravascular imaging modality which allows the differentiation of coronary tissue morphological characteristics including the identification of thin cap fibroatheroma and the differentiation between plaque rupture or erosion, due to its high resolution. In this narrative review we will discuss the role of OCT in patients presenting with MINOCA. In this group of patients OCT has been shown to reveal abnormal findings in almost half of the cases. Moreover, combining OCT with cardiac magnetic resonance (CMR) was shown to allow the identification of most of the underlying mechanisms of MINOCA. Hence, it is recommended that both OCT and CMR can be used in patients with a working diagnosis of MINOCA. Well-designed prospective studies are needed in order to gain a better understanding of this condition and to provide optimal management while reducing morbidity and mortality in that subset patients.

## 1. Introduction

Atherosclerotic cardiovascular disease is one of the leading causes of death around the world [1,2]. Advances in the understanding of the underlying pathobiology, diagnosis, and treatment of atherosclerosis have been made during the past century. This progress has significantly lowered the mortality rate in patients presenting with acute myocardial infarction (AMI) with obstructive coronary artery disease (CAD). However, in recent years challenges in the diagnosis and treatment of patients who presented with symptoms of AMI but did not have obstructive CAD have been recognized.

First reports of AMI without obstructive CAD go back 80 years [3,4]. This phenomenon was observed in the late 1970s by one of the pioneers in the field of interventional cardiology—DeWood. In his studies, he performed coronary angiography in patients presenting with ST elevation myocardial infarction (STEMI) and non-ST elevation myocardial infarction (NSTEMI) [5,6]. Surprisingly, about 10% of patients presenting with AMI had no significant CAD on coronary angiography. His initial observations were later confirmed in several large AMI registries in which 13% of patients presenting with AMI did not have obstructive CAD [7,8]. 

Important questions were raised regarding the underlying pathophysiological mechanism and treatment of this presentation. This led to the creation of new terminology for this phenomenon, myocardial infarction with non-obstructive coronary artery disease (MINOCA). The first position papers regarding MINOCA were published by the European Society of Cardiology (ESC) in 2018, followed by the American Heart Association in 2019 [9,10]. According to both position papers, the diagnosis of MINOCA should be made immediately upon coronary angiography in a patient presenting with features consistent with AMI [11]. Although chest pain and elevated troponin levels are not specific for AMI, MINOCA is an umbrella term for several different conditions, thus should only be a working diagnosis requiring further evaluation. MINOCA can be confirmed only after the investigation of other underlying causes of elevated troponin levels. Ascertaining the pathophysiological mechanism and prognostic markers in order to provide proper management strategies is vital in patients with a diagnosis of MINOCA. In this narrative review we will discuss the role of optical coherence tomography (OCT) in patients presenting with MINOCA.

## 2. Discussion

### 2.1. MINOCA: Is It a Serious Condition?

Significantly, patients presenting with MINOCA have comparable, or only a slightly lower, incidence of major adverse cardiac events (MACE) during follow-up as compared to those presenting with AMI, despite their younger age and less comorbidities [12,13]. 

Kang et al. showed that the 12-month MACE rate in patients with MINOCA was comparable to patients with AMI with single or double vessel CAD (7.8% vs. 12.2%; *p* = 0.359) (Table 1) [14]. Ishi et al. observed that MINOCA was associated with a high risk of in-hospital mortality compared with MI with obstructive CAD [15]. In a study which included 4793 consecutive patients presenting with STEMI, patients without obstructive CAD had a long-term risk of death similar to, or higher than, patients with obstructive CAD, although their causes of death were less often cardiovascular [16]. Lindahl et al., in a retrospective study involving almost 10,000 patients, observed a 13% mortality rate for MINOCA patients during four-year follow-up [17]. Gasior et al., in a retrospective study of over 6000 patients, demonstrated higher mortality at 12-month follow-up in the MINOCA group when compared to the MI-CAD group (10.94% vs. 9.54%, *p* < 0.001), with no statistical difference in mortality at three-year follow up [18].

Contrary to those findings, Pasupathy et al. in a metaanalysis including 28 publications, demonstrated that patients with MINOCA had lower one-year all-cause mortality [12]. However, it should be emphasized that even though patients with AMI had a higher one-year mortality rate at 6.7%, the mortality of patients with MINOCA was still high (4.7%). Considering that patients with stable chest pain (without previous AMI) and non-obstructed coronary arteries had 0.2% one-year mortality, the mortality in MINOCA patients was markedly elevated [22]. 

It is of the utmost importance to optimize the management of patients with MINOCA based on the underlying mechanism. Montone et al. observed that patients with vasospastic angina who had a reduction in their dosing of calcium channel blockers (CCB) had increased mortality during follow-up compared to those who continued to take high doses of CCB [23,24]. Of note, more than one third of patients with MINOCA did not receive an optimal cardioprotective pharmacotherapy [25,26]. 

### 2.2. Etiology of MINOCA

**Figure 1:**
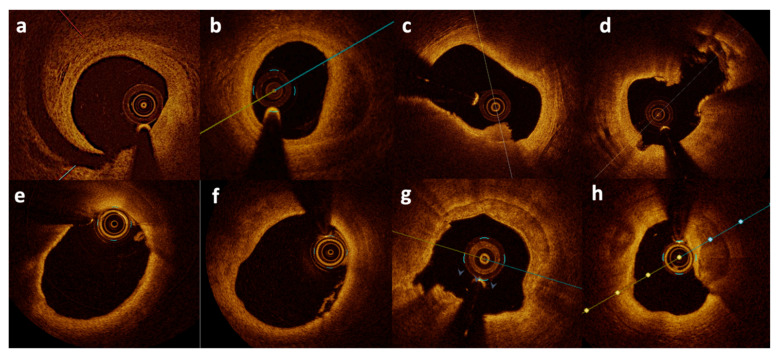
Optical coherence tomography images from patients with MINOCA. Spontaneous dissection (**a**,**b**), plaque erosion (**c**), plaque rupture (**d**), thin-cap fibroatheroma (**e**), small white thrombi (**f**), and calcified nodule erosion (**g**,**h**). Figures from authors’ library.

Myocardial infarction with non-obstructive coronary artery disease is a heterogenous entity with many possible etiologies that need to be clarified by proper diagnostics algorithm. Over the past several years, a few algorithms were developed in order to optimize the care of MINOCA patients [9,11,27]. Rigorous algorithms are crucial for effective treatment for certain conditions (for example, vasospasm) but may not be effective for another group of patients with MINOCA caused by a different mechanism (for example, plaque rupture) [11]. Common causes of a MINOCA working diagnosis may include plaque disruption, spontaneous coronary artery dissection (SCAD), coronary artery spasm, coronary thromboembolism, Takotsubo cardiomyopathy, and myocarditis. Importantly, due to the low resolution of coronary angiography, plaque disruption may occur in areas of coronary arteries which appear normal on the angiogram [28]. A large thrombus may result in severe narrowing or occlusion of the artery visible on angiogram, whereas smaller thrombi may either result in insignificant stenosis not visible on the angiogram or embolization to distal segments. Information regarding the exact pathogenic mechanism responsible for MINOCA, plaque vulnerability, or plaque burden cannot be obtained from angiography alone [29,30]. Spontaneous coronary artery dissection is another diagnosis which cannot be completely ruled out with angiography alone [31]. Two intravascular imaging modalities have been proposed to surpass the limitations of angiography: intravascular ultrasound (IVUS) and optical coherence tomography (OCT). IVUS studies showed that plaque rupture or ulceration may be identified in about 40% patients presenting with MINOCA [32,33]. Optical coherence tomography with a resolution of 10–20μm allows the visualization of intraluminal and superficial coronary artery structures in detail [34]. It has the ability to differentiate tissue morphological characteristics including the detection of lipid-rich, calcified, and fibrous plaques, thin cap fibroatheroma, and the differentiation between plaque rupture and erosion, red and white thrombi, as well as the identification of even small spontaneous dissections (Figure 1) [35,36]. It can function as a type of optical biopsy and is a powerful imaging technology for medical diagnostics. Unlike conventional histopathology, which requires removal of a tissue specimen and processing for microscopic examination, OCT can provide images of the vascular wall in situ and in real time. Its higher resolution undoubtedly can confirm findings such as plaque erosion or calcified nodule which may cause AMI and usually are not visible on both conventional angiography and IVUS. 

Howbeit, it should be emphasized that OCT also has several drawbacks [37]. First, its greater resolution as compared to IVUS comes with a lower penetration depth. In the case of large arteries such as the left main, visualization of the whole coronary artery may not be possible. Moreover, when performing pullback in ostial lesions incomplete blood clearance may lead to suboptimal image quality. Second, the need for contrast agents to clear blood may increase risk of contrast-induced nephropathy. Third, OCT images cannot penetrate lipid plaque and red thrombi. 

According to a recent metaanalysis, up to 33% of patients with the diagnosis of MINOCA may have myocarditis [38]. In a recent prospective study, cardiac magnetic resonance (CMR) showed evidence of myocarditis in 25% of patients presenting with MINOCA, an MI in 25%, and cardiomyopathy in 25% [39]. Recent studies demonstrated the value of combined CMR and OCT imaging in MINOCA patients. Moreover, it should be stressed that finding one cause of MINOCA does not necessarily mean that others have been excluded. Several studies have emphasized the importance of coronary artery vasospasm in Takotsubo cardiomyopathy and myocarditis [40,41]. An OCT study including 23 patients found that those with Takotsubo cardiomyopathy have high plaque vulnerability [42].

### 2.3. OCT in MINOCA

Coronary thrombosis is the most frequent final event leading to an acute coronary syndrome in patients with AMI with obstructive coronary disease. Plaque rupture, plaque erosion, and calcified plaque are believed to be the most common underlying mechanisms contributing to AMI with the former being the most frequent in both autopsy and in vivo studies [43,44]. 

While angiographic images of haziness or minor filling may suggest plaque disruption, it can be definitively diagnosed using intracoronary imaging, with OCT being the preferable modality due to its higher resolution. However, IVUS may be considered as an alternative to OCT to a lesser extent [45]. One of the first OCT studies in MINOCA patients showed that plaque disruption or thrombi were visible in 39% of 38 patients included in the study [46] (Table 2). Notably, during hospitalization 82% patients underwent CMR. In a detailed assessment of infarct-related arteries (i.e., those where infarct-related artery was identified on the basis of the association between coronary artery distribution and myocardial segments with late gadolinium-enhancement of ischemic origin), the authors found that 40% had plaque rupture and 30% had plaque erosion. Importantly, 30% of lesions had plaque disruption without thrombus. The latter might have resulted, as stated by the authors, either by resolution of thrombi from the initial antithrombotic therapy or by distal embolization during advancement of the OCT catheter. It could have also been an incidental finding after silent plaque rupture which occurred in the near past [47]. Findings by Opolski et al. led to the modification of the initial treatment in six patients. One of the main limitations of this study was the relatively small number of patients recruited which could present bias. Moreover, only 21% of the patients had three-vessel OCT. 

In a small study by Mas-Lladó et al. involving 27 patients with MINOCA who had mostly one-vessel OCT, an abnormal image was found in 78% of patients [48]. Patients predominantly had either plaque erosion (41%) or plaque rupture (30%). 

In a more recent study presented by Gerabaud et al. 40 patients with MINOCA underwent both OCT and CMR [50]. Optical coherence tomography provided a diagnosis of AMI in 80% of patients including 35% with plaque rupture, 30% with plaque erosion, 7.5% with lone thrombus, 5% with SCAD, and 2.5% with calcified nodule. Acute myocardial infarction was evident in CMR in 77.5% of patients. Over half the patients (57.5%) had a substrate and/or diagnosis supported by both modalities, 22.5% of patients had a mechanism specified only by OCT, and 20% of patients had a clear diagnosis only by CMR. One of the major findings of this study was that combination of both CMR and OCT provided a much higher yield in diagnosing MINOCA as compared to using only one of the mentioned modalities. The limitations of this study were similar to the study of Opolski et al.—the small number of patients and the low number of patients with three-vessel OCT (12.5%). Moreover, OCT was not always done at the index procedure, and an older CMR imaging protocol was used. 

Reynolds et al. presented the biggest study to date, involving 145 women with a diagnosis of MINOCA [49]. In this study CMR was interpretable in 116 patients. Over half of the patients had three-vessel OCT (59.3%) and a possible culprit lesion was identified in 46.2% of patients. Plaque rupture, intra-plaque cavity, or a layered plaque phenotype were evident in 39% of patients, whereas thrombus without plaque rupture was found in 3.5% of patients and one patient had SCAD. Moreover, 2.1% of patients had intimal bumping suggestive of coronary artery spasm. Combining both OCT and CMR allowed the identification of the cause of MINOCA in 84.5% of patients. A lesion visible on OCT could be identified in 42% of patients with CMR-detected infarction and in 79% of patients with CMR-detected regional injury. Hypothetically, patients who had CMR evidence of infarction or regional injury without abnormalities identified by OCT could suffer from coronary spasm or thromboembolism as the mechanism of MI. Importantly, 40% of patients without abnormal CMR had an OCT identified culprit lesion—this finding underlines the importance of OCT in the diagnosis of MINOCA and strengthens the guidelines which suggest multimodality imaging in patients with MINOCA. Reynolds et al. confirmed previous findings that multi imaging modalities, including both OCT and CMR, should be used in patients with MINOCA—the identification of the etiology of MINOCA may have potential to guide optimal medical therapy; however, new studies are warranted. Limitations of this study were the lack of three-vessel OCT in all patients and the inclusion of layered plaque phenotype and intra-plaque cavity as causes of MINOCA. Layered plaque phenotype is a consequence and not an etiology of plaque destabilization. The process of lesion progression to a layered plaque phenotype may take from weeks to months. Moreover, a recent OCT study reported that a layered plaque phenotype may be found in more than 50% of patients with stable angina [51]. To our knowledge, there was only one case report for the OCT finding of intraplaque hemorrhage. It should be emphasized that there is a difference in methodology used for OCT interpretation between the presented studies. Some studies include lone thrombus which in these authors’ opinion may not always be easy to distinguish from plaque erosion. Also, other definitions were introduced, such as layered plaque phenotype. This may cause differences in incidence of OCT findings between different studies. 

Although pathogenesis of SCAD remains unclear there is some evidence that it is related to connective/collagen tissue alterations. In-hospital mortality of patients with SCAD is similar to those with obstructive CAD. On angiogram, SCAD may be missed or misdiagnosed as vasospasm due to low resolution of the image, even though there may be a life-threatening condition [52]. In the recent OCT and CMR study, the incidence of SCAD was up to 5%. It is therefore crucial to perform both OCT and CMR in patients with a working diagnosis of MINOCA [53]. 

Coronary artery spasm reflects a vascular smooth muscle hyper-reactivity to endogenous vasospastic substance, but may also occur in the context of exogenous vasospastic agents [11,54]. Prevalence of coronary artery spasm in patients with MINOCA may vary between 3% and 95% [55]. Moreover, previous studies have shown that about one quarter of the patients with MINOCA have evidence of microvascular spasm [56]. In a recent study, Montone et al. showed that out of 80 enrolled patients presenting with MINOCA, a provocative test was positive in almost half of the patients [23]. Furthermore, a thrombus was found by OCT in 28.8% of patients presenting with vasospastic angina [57]. In patients presenting with vasospasm-induced AMI intimal tear, intra luminal thrombi and plaque erosion were significantly more frequent compared to patients with chronic stable vasospastic angina [58]. Thus, OCT may be a useful modality when assessing MINOCA patients suspected for coronary artery spasm. Coronary artery spasm on OCT is characterized by intimal bumping with a larger medial area and medial thickness [59]. 

**Figure 2:**
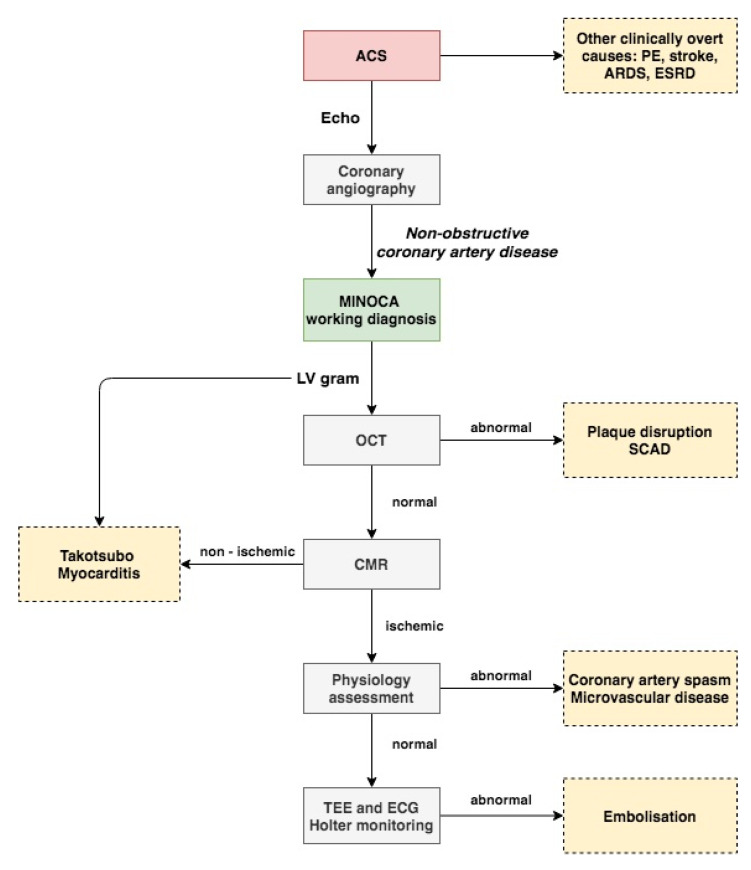
Proposed approach to myocardial infarction with non-obstructive coronary artery disease diagnosis. Flowchart is explained in the text. ACS indicates acute coronary syndrome; ARDS, acute respiratory distress syndrome; CMR, cardiac magnetic resonance; ESRD, end stage renal disease; MINOCA, myocardial infarction with non-obstructive coronary artery disease; OCT, optical coherence tomography; SCAD, spontaneous coronary artery dissection; and TEE, trans esophageal echocardiography.

Most of the current studies support the necessity of OCT in the diagnosis of patients presenting with MINOCA. Proper management of every patient with suspected myocardial infarction should include several different imaging modalities. A proposed approach to the proper diagnosis of patients with MINOCA is presented in Figure 2. In the authors’ opinion, the first step starts with proper analysis of trans thoracic echocardiography (TTE) performed before angiography. Next, during coronary angiography when MINOCA is identified, angiography of the left ventricle (LV) could be of help for assessment of regional wall abnormalities. A combination of both TTE and LV angiography could be used to identify Takotsubo cardiomyopathy or myocarditis. OCT can be used to evaluate coronary arteries based on findings in the electrocardiogram, TTE, or LV angiography. If no abnormalities, such as plaque disruption or SCAD, are found on OCT, CMR should be performed [60]. Finally, other tests such as the intracoronary acetylcholine provocation test could be considered for further evaluation of MINOCA patients in order to identify abnormalities, such as coronary artery spasm or microvascular dysfunction. 

## 3. Conclusions

Although AMI and non-obstructive coronary artery disease have been known for more than five decades, our knowledge is limited and many challenges still remain. Current studies show the importance of using OCT and CMR in patients with a working diagnosis of MINOCA. Moreover, when no abnormal findings are present on OCT, other tests should be performed in order to assess the coronary flow reserve (CFR) and microcirculatory resistance (iMR). Although recent studies shed light on the pathogenesis of MINOCA, well-designed prospective studies are needed in order to gain a better understanding of this condition and to provide optimal management while reducing morbidity and mortality in patients with MINOCA.

## Figures and Tables

**Table 1:** Selected studies with outcomes of patients with myocardial infarction with non-obstructive coronary artery disease.

Study	No. of Patients with Non-Significant CAD, *n*	No. of Patients with Significant CAD, *n*	Follow-Up Length	Mortality	MACE	Other	STEMI at Admission	Notes
Safdar et al. [19]	<50% CAS-299	≥50% CAS-2374	12 months	1 month: 1.1% vs. 1.7% (*p* = 0.43) 12 months: 0.6% vs. 2.3% (*p* = 0.68)	NA	SAQ: 76.5 vs. 73.5 (*p* = 0.06)	21.4% vs. 52.1% *p* = 0.001	NA
Kang et al. [14]	<50% CAS-372 (^A^)	>50% CAS (one or two-vessel disease)-6136 (^B^) >50% CAS (three-vessel disease or LM disease)-2002 (^C^)	12 months	In hospital: 2.2% (^A^) vs. 2.6% (^B^) vs. 6.9% (^C^); *p* = 0.952 (^A^ vs. ^B^).	12 months 7.8% (^A^) vs. 12.2% (^B^) vs. 23.3% (^C^); *p* = 0.359 (^A^ vs. ^B^).	Repeat PCI at 12 months: 2.4% (^A^) vs. 2.4% (^B^) vs. 14.0% (^C^); *p* = 0.180 (^A^ vs. ^B^).	36.3% (^A^) vs. 63.8% (^B^) vs. 52.0% (^C^); *p* < 0.001 (^A^ vs. ^B^).	NA
Ishii et al. [15]	<50% CAS-14,045	≥50% CAS-123,633	30 days	In hospital: 6.4% vs. 6.2%	NA	NA	NA	NA
Andersson et al. [16]	Normal CA-256 (^A^) Non-obstructive CAS-298 (^B^)	≥50% CAS-4239 (^C^)	2.2 years	CVD 3.5% (^A^) vs. 5.0% (^B^) vs. 9.8% (^C^) Non-CVD 7.4% (^A^) vs. 8.4% (^B^) vs. 4.2%	NA	NA	NA	NA
Lindahl et al. [17]	9466 (9136 after one month)		4.1 years	13.4%	23.9%	NA	17.1%	NA
Larsen et al. [20]	<30% CAS-127	≥30% CAS-3475	3 years	CVD 0.8% vs. 4.0% (*p* = 0.12)	7.7% vs. 22.2% (*p* = 0.002)	Re-infarction: 0% vs. 1.9% (*p* = 0.12)	100%	Study included only patients with STEMI
Gasior et al. [18]	<50% CAS-6063	>50% CAS-160886	36 months	16.8% vs. 14.93% (*p* = 0.081)	NA	PCI at 36 months: 5.82% vs. 23.9% (*p* < 0.01)	NA	NA
Grodzinsky et al. [21]	≤70% CAS-381	>70% or >50% in LM CAS-4941	12 months	3.9% vs. 3.1% (*p* = 0.08)	NA	Angina prevalence at 12 months: 24.6% vs. 21.4% (*p* = 0.199) SAQ QOL 60.5 vs. 63.8 (*p* = 0.006)	13.4% vs. 49.0% (*p* < 0.001)	NA

CA indicates coronary artery; CAS, coronary artery stenosis; CVD, cardiovascular disease; LM, left main; MACE, major adverse cardiovascular events; NA, not available; PCI, percutaneous coronary intervention; SAQ, Seattle Angina Questionnaire; STEMI, ST-elevation myocardial infarction; and QOL, quality of life. ^A^, ^B^ and ^C^ stand for different groups. When not indicated, results of patients with myocardial infarction with non-obstructive coronary artery disease are given first.

**Table 2:** Myocardial infarction with non-obstructive coronary artery disease selected studies with use of optical coherence tomography.

Study	No. of Patients	Modalities Used, *n*	Three-Vessel OCT, *n*	Two-Vessel OCT, *n*	Abnormal Image in OCT, *n*	Plaque Rupture, *n*	Plaque Erosion, *n*	Calcified Nodule, *n*	Lone Thrombus, *n*	SCAD, *n*	Other, *n*	Abnormal Image in CMR, *n*	Abnormal Image OCT or CMR, *n*
Opolski et al. [46]	38	OCT-38 (100%) CMR-31 (82%)	8 (21%)	26 (68%)	15 (39%)	8 (21%)	4 (11%)	2 (11%)	2 (5%)	NA	Takotsubo-5 (13%) Myocarditis-3 (8%)	16 (52%) *	NA
Mas-Lladó et al. [48]	27	OCT-27 (100%)	0	1 (4%)	21 (78%)	8 (30%)	11 (41%)	2 (7%)	NA	NA	NA	NA	NA
Gerbaud et al. [38]	40	OCT-40 (100%) CMR-40 (100%)	5 (13%)	11 (28%)	32 (80%)	14 (35%)	12 (30%)	1 (3%)	3 (8%)	2 (5%)	NA	31 (78%)	40 (100%)
Reynolds et al. [49]	145	OCT-145 (100%) CMR-116 (80%)	86 (59%)	47 (32%)	67 (46%)	8 (6%)	NA	0 (0%)	Thrombus without plaque rupture-5 (4%)	1 (1%)	Intra plaque cavity-31 (21%) Layered plaque-19 (13%) Intimal bump-3 (2%)	86 (74%)	98 (85%)

CMR indicates cardiac magnetic resonance; OCT, optical coherence tomography; SCAD, spontaneous coronary artery dissection; and TCFA, thin cap fibroatheroma. * T1-weighted imaging.
